# Endovascular embolization of spontaneous rupture of isolated splenic artery dissection associated with hemosuccus pancreaticus: a case report

**DOI:** 10.1186/s12872-021-02148-6

**Published:** 2021-07-09

**Authors:** Jianjun Jiang, Yang Liu, Xiangjiu Ding

**Affiliations:** 1 Department of Vascular Surgery, General Surgery, Qilu Hospital of Shandong University, 107 Wenhua Xi Road, Jinan, 250012 People’s Republic of China; 2 Department of Pharmacology, School of Basic Medical Sciences, Shandong University, Jinan, 250012 People’s Republic of China

**Keywords:** Splenic artery, Dissection, Rupture, Hemosuccus pancreaticus, Case report

## Abstract

**Background:**

Isolated splenic artery dissection (SAD) is extremely rare, life-threatening, and particularly difficult to diagnose. Moreover, SAD presenting as digestive hemorrhage has not been reported.

**Case presentation:**

A 44-year-old man presented with recurrent life-threatening hematochezia. Magnetic resonance and computed tomographic angiography showed isolated SAD with an intrapancreatic hematoma. Selective angiography confirmed the diagnosis of rupture of SAD. Hemosuccus pancreaticus was considered the potential mechanism of digestive hemorrhage. It was successfully managed by endovascular coil embolization.

**Conclusions:**

Isolated SAD is especially rare but fatal. Rupture of SAD should be considered in the differential diagnosis as a rare cause of digestive hemorrhage. Endovascular coil embolization is effective in treating ruptured SAD.

## Background

Splenic artery dissection (SAD) usually originates from dissection of the celiac trunk or the abdominal aorta [1]. Isolated SAD is extremely rare but life-threatening [2]. To date, only eight cases with isolated SAD have been reported in literature and six (75%) had dissection rupture. Of these six cases, five died and only one was successfully treated by open surgery [2]. Ruptured SAD usually presents with abdominal pain and results in massive retroperitoneal or intraperitoneal hemorrhage [2]. However, to our knowledge, it has not been reported that ruptured SAD presents with painless hematochezia. Herein, we present a case of spontaneous rupture of isolated SAD presenting as recurrent, life-threatening digestive hemorrhage.

## Case presentation

**Fig. 1:**
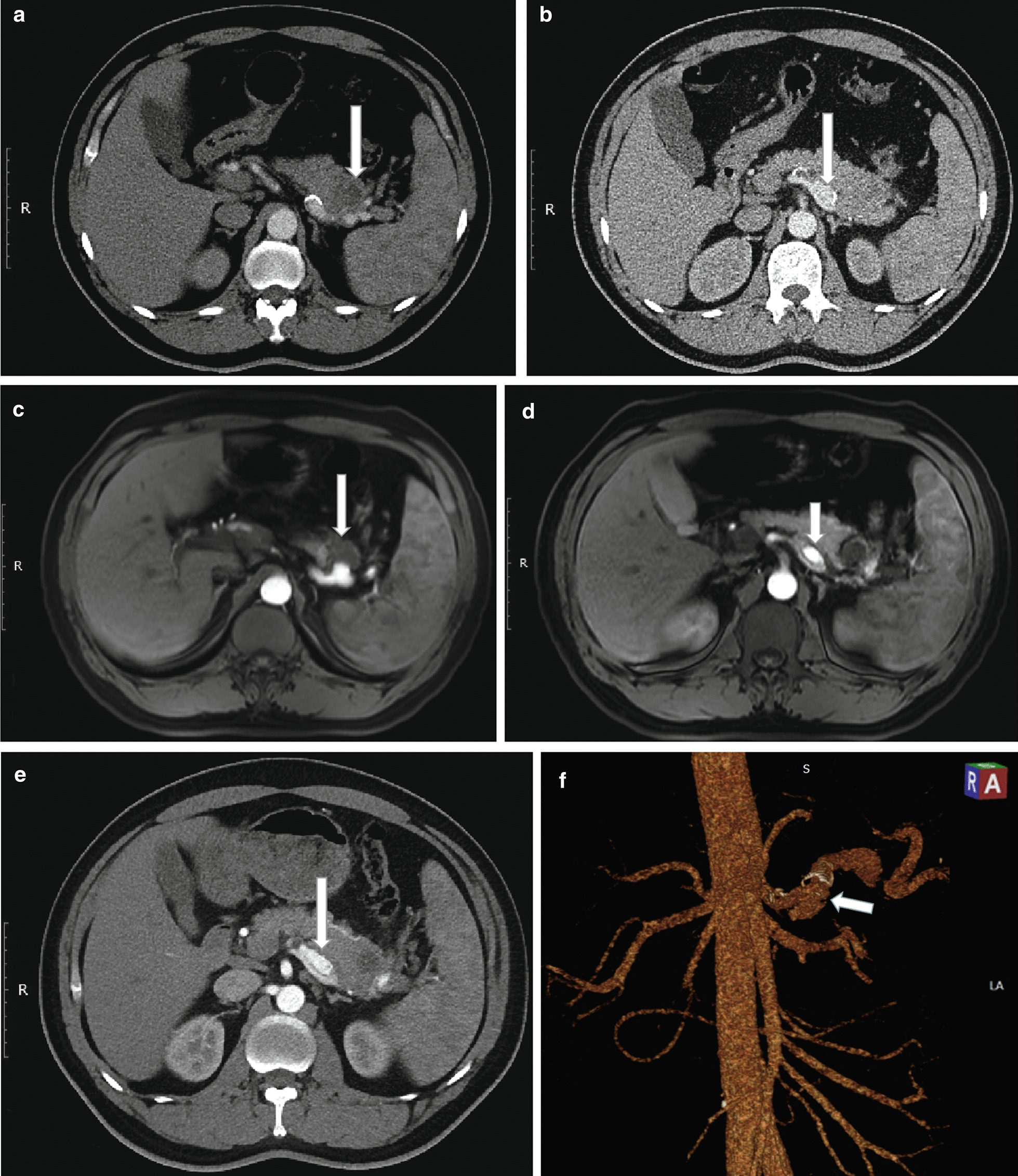
Preoperative images: **a** Contrast-enhanced computed tomography (CT) reveals a 3.6 × 3.4 cm^2^ intrapancreatic hypodense mass (white arrow) close to the splenic artery (SA). **b** CT shows dilation of the calcified SA (white arrow). **c** Magnetic resonance angiography shows a hematoma (white arrow) close to the protruding SA. **d** The proximal SA is divided into two lumens. **e**, **f** CT Angiography confirms isolated splenic artery dissection (SAD) with a typical “double-lumen” sign (white arrows). The SA was partially thrombosed in the proximal segment

A 44-year-old man was referred from a local hospital with a 30-day history of intermittent hematochezia. No abdominal pain was noted, and his physical examination was unremarkable. His medical history included a 6-month history of hypertension, no histories of abdominal trauma, surgery, or pancreatitis, and no use of nonsteroidal anti-inflammatory drugs. Laboratory examinations, including tumor markers, were within normal limits, except for anemia (hemoglobin: 77 g/L [normal: 120–160]). Esophagogastroduodenoscopy, colonoscopy, and capsule endoscopy were performed, but these procedures failed to find the source of the bleeding. Contrast-enhanced computed tomography (CT) showed a 3.6 × 3.4 cm^2^ hypodense mass (Fig. 1a, white arrow) which was suspected to be a neoplasm and a dilated splenic artery (Fig. 1b, white arrow). Magnetic resonance (MR) angiography demonstrated that the mass was an intrapancreatic hematoma (Fig. 1c, white arrow) close to the protruding splenic artery, which was separated into two lumens at the proximal segment (Fig. 1d, white arrow). CT angiography confirmed isolated dissection of the splenic artery with a typical “double-lumen” sign (Fig. 1e, f, white arrows).

**Fig. 2:**
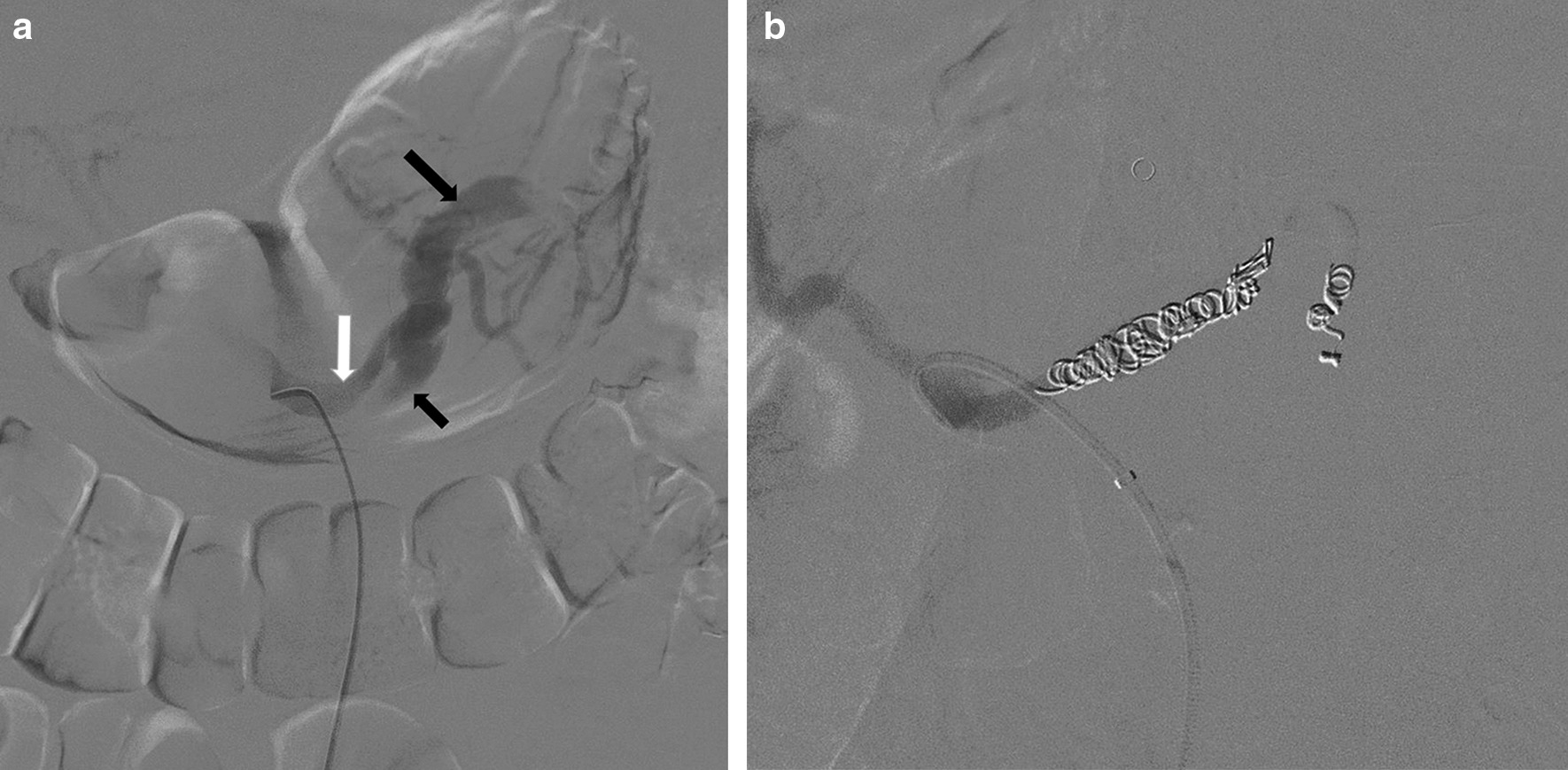
Intraoperative images: **a** Selective SA angiogram demonstrates the true (white arrow) and false (black arrows) lumens of SAD. The distal false lumen (large black arrow) obviously dilated and deviated from the true lumen. **b** Angiogram of the celiac trunk shows the SA is occluded by coils

Hematochezia recurred eight hours after the patient was admitted to our hospital. The patient suffered from hemorrhagic shock and received fluid resuscitation and blood transfusions. A diagnosis of ruptured SAD was considered. Digital subtraction angiography was performed emergently. The right femoral artery was percutaneously punctured. A 6 F, 50-cm-long sheath (Cook Inc, Bloomington, IN) was introduced over a 0.035-in., 150-cm guidewire. Selective superior and inferior mesenteric artery angiography procedures were unremarkable. Celiac artery angiography via a 5 F Cobra catheter confirmed the true (Fig. 2a, white arrow) and false (black arrows) lumens of SAD. The distal false lumen (large black arrow) was obviously dilated and deviated from the true lumen, which was consistent with the site of the hematoma on CT and MR. Spontaneous rupture of isolated SAD was diagnosed. Subsequently, endovascular embolization was performed. A 2.6-F, 125-cm-long microcatheter (Asahi Intecc, Nagoya, Japan) with a 0.018-in., 180-cm-long microguidewire (Asahi Intecc) was introduced through the Cobra catheter into the distal splenic artery. Both proximal and distal ends of the lesion were embolized via the microcatheter with ten Tornado microcoils (Cook Inc, Fig. 2b).

**Fig. 3:**
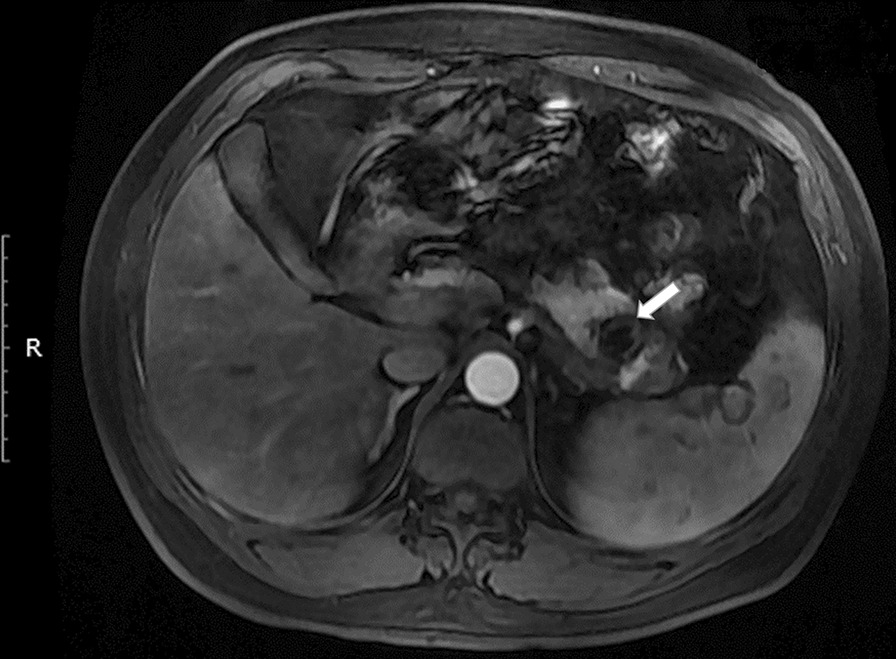
Contrast-enhanced magnetic resonance image at 2-month follow-up shows the occluded main trunk of the SA and a chronic hematoma (white arrow)

The patient recovered uneventfully after endovascular treatment. Contrast-enhanced MR at the two-month follow-up showed the occluded main trunk of the splenic artery with a chronic hematoma (Fig. 3, white arrow). The patient has been followed up for one year, and there was no recurrence of digestive hemorrhage. Written informed consent was obtained from the patient for publication of this case report.

## Discussion and conclusion

To the best of our knowledge, this is the first case of isolated SAD which was successfully treated by endovascular therapy. Although the etiology of SAD remains unclear, hypertension is considered to be a common risk factor [2]. This is consistent with the history of hypertension in the current case. Ruptured SAD usually presents with upper abdominal or left flank pain and causes intraperitoneal or retroperitoneal hemorrhage [2]. However, in the current case, it presented with intermittent hematochezia and resulted in digestive hemorrhage. To our knowledge, SAD resulting in digestive hemorrhage has not been reported in literature. Herein, we present the first case of SAD presenting as recurrent digestive hemorrhage. Ruptured SAD should be considered in the differential diagnosis as a rare cause of digestive hemorrhage.

Splenic artery diseases rarely result in digestive hemorrhage. Most of them are false splenic artery aneurysms (SAAs) caused by pancreatitis or pancreatic tumors [3–7]. SAAs can rupture into the stomach or the pancreatic duct and lead to gastric or duodenal hemorrhage [3–7]. The latter hemorrhage is also termed “hemosuccus pancreaticus” [4, 6, 7]. The diagnosis of hemosuccus pancreaticus remains challenging. It can be difficult to identify the bleeding site via endoscopy or digital subtraction angiography because of its rarity, anatomical location, and intermittent characteristics [4, 7]. Endoscopy was able to detect active bleeding via the papilla in only 30% of cases [7].

In this case, endoscopies failed to identify the bleeding site, and histopathological examination was not performed because of endovascular treatment. However, the authors believe that digestive hemorrhage was caused by rupture of SAD. The potential mechanism is considered to involve hemosuccus pancreaticus: SAD ruptured into the pancreatic tail, and the blood from the hematoma entered into the duodenum via the pancreatic duct. This is supported by several observations. First, the hematoma was completely within the pancreas. The site is similar to Yoshikazu’s cases [6]. The SAAs observed in their cases were treated by open surgery, and the communication between the aneurysm and the pancreatic duct was proven by histological examination. Second, hemosuccus pancreaticus is usually intermittent and repetitive [4, 7]. These characteristics were consistent with the present case. Third, primary gastrointestinal or pancreatic lesions were excluded via endoscopies (gastroduodenoscopy, colonoscopy, and capsule endoscopy) and radiological examination (digital subtraction angiography, repeated CT, and MR). SAD rupturing into the stomach was also excluded based on the symptoms (hematochezia rather than hematemesis), endoscopies, and the range of the lesion, which did not exceed the superior edge of the pancreas on CT and MR. Finally, digestive hemorrhage was cured after the use of endovascular coil embolization for SAD. The efficacy has been demonstrated by 1-year follow-up.

Several methods have been reported in the treatment of SAAs, including open resection, laparoscopic resection, and endovascular treatment. For hemosuccus pancreaticus associated with SAAs, if the source of the bleeding is identified on angiography, endovascular treatment should be considered as the first choice of treatment [4, 6, 7]. Surgical treatment remains an important supplement if angiography fails to identify the source of the bleeding or if endovascular treatment is not successful. For SAD, only one patient was successfully managed with open surgery [2]. In the current case, it was successfully treated by endovascular coil embolization. Endovascular treatment is a safe and effective option to ruptured SAD.

In conclusion, isolated SAD is extremely rare but life-threatening. Ruptured SAD should be considered in the differential diagnosis as a rare cause of digestive hemorrhage. Selective angiography is recommended for diagnosis, and endovascular embolization is a safe and effective treatment for ruptured SAD.

## Data Availability

All data generated or analyzed during this study are included in this published article.

## Abbreviations

SAD: Splenic artery dissection
CT: Computed tomography
MR: Magnetic resonance
SAAs: Splenic artery aneurysms
SA: Splenic artery
